# Building a secreting nanomachine: a structural overview of the T3SS^☆^

**DOI:** 10.1016/j.sbi.2013.11.001

**Published:** 2014-04

**Authors:** Patrizia Abrusci, Melanie A McDowell, Susan M Lea, Steven Johnson

**Affiliations:** Sir William Dunn School of Pathology, Oxford University, South Parks Road, Oxford OX1 3RE, United Kingdom

## Abstract

To fulfill complex biological tasks, such as locomotion and protein translocation, bacteria assemble macromolecular nanomachines. One such nanodevice, the type III secretion system (T3SS), has evolved to provide a means of transporting proteins from the bacterial cytoplasm across the periplasmic and extracellular spaces. T3SS can be broadly classified into two highly homologous families: the flagellar T3SS which drive cell motility, and the non-flagellar T3SS (NF-T3SS) that inject effector proteins into eukaryotic host cells, a trait frequently associated with virulence. Although the structures and symmetries of ancillary components of the T3SS have diversified to match requirements of different species adapted to different niches, recent genetic, molecular and structural studies demonstrate that these systems are built by arranging homologous modular protein assemblies.

## Highlights

• Flagellar and non-flagellar T3SS are built assembling homologous protein machineries.
• Unified nomenclature for non-flagellar T3SS.
• New model of the T3SS needle is consistent with the flagellar filament, both in terms of helical parameters and orientation.
• Structural and functional implication of the new architecture of the T3SS export apparatus and ATPase complex.

## Graphical abstract

**Figure:**
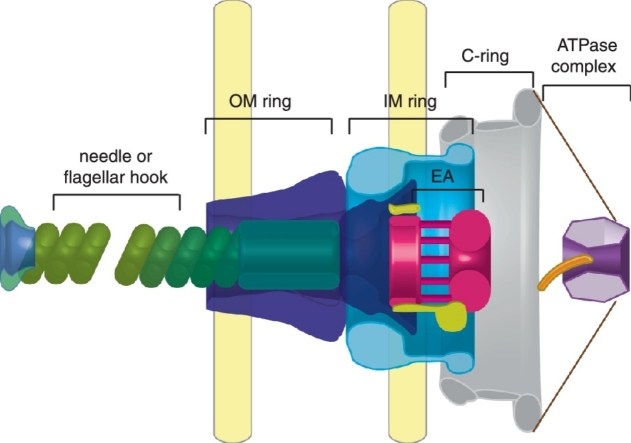

**Current Opinion in Structural Biology** 2014, **25**:111–117

This review comes from a themed issue on **Macromolecular Machines**

Edited by **Karl-Peter Hopfner** and **Tom Smith**

For a complete overview see the Issue and the Editorial

Available online 1st April 2014

0959-440X/$ – see front matter, © 2014 The Authors. Published by Elsevier Ltd. All rights reserved.

**http://dx.doi.org/10.1016/j.sbi.2013.11.001**

The core of both flagellar-and NF-T3SSs shows an evolutionary related architecture [1] consisting of a multi-ring basal structure embedded in both inner and outer bacterial membranes, with its proximal end connected to an export apparatus (EA) and to an ATPase complex in the cytosol. The T3SS is welded at its distal end to a needle to translocate virulence proteins directly into the host or a flagellar hook onto which polymerizes an extracellular filament dedicated to locomotion [2,3] (Figure 1a). In this review we focus on the recent breakthroughs about the supramolecular structure of NF-T3SS key subassemblies, highlighting analogies to the equivalent flagellar components, when appropriate. For clarity, we will refer to the proteins of the NF-T3SS according to their secretion and cellular translocation (Sct) abbreviation (Table 1), a unified naming system previously proposed [4].

One area in which major strides have been taken in the last two years is in the study of the needles of the NF-T3SS, the major extracellular component formed by the helical assembly of multiple copies of a single protein (SctF) in an analogous manner to the assembly of the flagellar hook and filament [5]. However, the small size of SctF (∼9 kDa), combined with the natural propensity toward polymerization, has made high resolution structural studies challenging. Initial studies on the *Shigella flexneri* needle by negative stain EM demonstrated that it shared similar helical parameters (∼5.5 subunits/turn; 4.6 Å axial rise/subunit) to the flagellar hook/filament [6]. Subsequently, X-ray crystallography and NMR [7–13] showed that SctF consisted of a conserved two helix coiled-coil fold, similar to the filament building D0 domains of the flagellar components. However, fitting of an X-ray crystallographic structure of the monomer into a 16 Å EM map produced a model for the needle in which the N-terminal helix lined the channel [7], at odds with the orientation of the D0 helices of the flagellar filament model built in a 4–5 Å cryo-EM map. Further confusion was introduced with the publication of two independent studies of the *Salmonella typhimurium* needle [14]. The first, using cryo-EM analyses, suggested very different helical parameters (∼6.3 subunits/turn) to the *S. flexneri* needle and *S. typhimurium* flagellum. The second, using a combination of techniques, suggested that a double point mutant of *S. typhimurium* SctF underwent a structural rearrangement upon polymerization, whereby the C-terminal 25% of the protein went from α-helical to β-strand, again at odds with the polymerization of the flagellum [9].

Several elegant high resolution studies have now gone a long way toward resolving these various inconsistencies. Early in 2012 a 7.7 Å cryo-EM map of the *S. flexneri* needle confirmed the flagellum-like parameters and demonstrated that 90% of the protein was α-helical [15^•^]. Interestingly a novel, non-helical protrusion was observed and based on this the authors proposed an alternative model for the *S. flexneri* needle in which residues 51-65 of SctF were remodeled from the α-helix seen in the high resolution structures to a β-hairpin structure (Figure 2a). Shortly after this, a new needle architecture was proposed for the *S. typhimurium* needle [16^••^]. Using a combination of solid state NMR and Rosetta modeling the authors produced a needle in which the C-terminal helix of SctF lines the needle channel, i.e., opposite to the previous models (Figure 2b). This orientation was validated by immuno-EM and was subsequently confirmed for the *S. flexneri* needle [17^•^]. Gratifyingly, the new model also fits well to the *S. flexneri* cryo-EM map, with the non-helical protrusion likely to be explained by the extended N-terminus (Figure 2c). These studies therefore return the NF-T3SS needle to being consistent with the flagellar filament, both in terms of helical parameters and orientation, with the highly conserved C-terminal helix lining the channel and forming the majority of the inter-subunit connections.

As well as demonstrating the power of combining multiple high resolution structural techniques, this new needle model has implications for the structures of the periplasmic rod and distal tip assemblies. Structural information regarding the rod component SctI is scant, with NMR studies showing that the monomeric protein is mostly unfolded in solution with a 15 residue stretch of α-helix in the C-terminal half [18^•^]. However, this combined with the homology to the needle monomer in the C-terminal helix suggests a model for the rod being assembled in much the same way, with the more variable N-terminal half decorating the outside. At the other end of the needle, despite a plethora of high resolution structures of tip protein monomers, confusion reigns over the structure of the tip assembly. A number of models have been proposed based on low resolution EM projections/maps and crystal structures [10,19,20], but they suffer from a lack of consistency even at the most basic level as to whether they display the helical symmetry of the needle or pure rotational symmetry. This is clearly another area where higher resolution information is required.

More recently, evidence from both high and low resolution structural techniques has demonstrated an overall threefold symmetry for the NF-T3SS, with an either 12-fold [21] or 15-fold [22^••^] outer membrane (OM) secretin ring through a 24-fold inner membrane (IM) ring [22^••^] to the 9-fold of the major component of the EA [23^••^] which is functionally coupled to the hexameric ATPase [24,25,26^••^]. Despite three-dimensional reconstructions from negative stain and cryo-EM, together with top views of selective disassembled basal body rings of isolated *S. typhimurium* and *Yersinia enterocolitica* T3SS, the symmetry of the OM ring remains ambiguous with genuine inter-species differences proposed as one resolution of the conflicts between different symmetric arrangements of SctC [21,22^••^,27]. However, the IM ring, which was previously debated as being 12-fold, 20-fold or 24-fold symmetric [28–30] has been revealed to be two concentric rings, each made of 24 subunits (the integral bitopic SctD outside and the lipidated SctJ inside) (Figure 1b [22^••^]). Crosslinking and interface disruptive mutations also support some of the proposed symmetries and ring interactions [22^••^,31,32]. All three of the proteins that dominate the periplasmic regions are modular and contain one or more copies of two ‘ring forming motifs’ (α-β-β-α-β or β-α-β-β-α), through which the backbone of each ring is built [31,33]. More recently, the *in situ* tomographic reconstruction of the *Y. enterocolitica* T3SS, and the crystal structure of the equivalent SctD periplasmic domains [34^•^], seem to suggest that both the OM and IM components are capable of stretching vertically along the axis of the basal body. However the functional implications of such a mechanism are yet to be resolved. Understanding the molecular details of the symmetry mismatch in these assemblies and its effect on the formation and disruption of a web of non-covalent interactions among subunits is a crucial step to learn how the design of these nanomachines conjugates plasticity and integrity of the holostructure with its efficient assembly [35,36^••^].

Electron density maps also reveal that the SctD ring in the periplasmic region is less mobile than the ring formed by the SctD N-terminal cytosolic domain (SctD-N). The structure of this domain has been recently determined for several organisms [31,37,38^•^,39,40], revealing a forkhead-associated (FHA) fold with 4-stranded and 5-stranded β-sheets packing against each other in a globular structure that is conserved despite the low sequence identity amongst family members. However, all homologues lack the full-repertoire of highly conserved residues required for phospho-threonine binding, suggesting that any interactions with these FHA domains are occurring in a phosphorylation-independent manner. Although one study reported an interaction between *S. flexneri* SctD-N and phosphorylated peptides [39], other studies indeed indicate that these domains are unable to bind phosphorylated substrates and thus are likely to function as non-canonical FHA domains [31,37,38^•^,40].

Although a NF-T3SS C-ring has not yet been imaged, molecular evidence and sequence homology strongly suggest the existence of a structure analogous to the flagellar C-ring beneath the basal body [41]. This has been postulated to be involved in secretion substrate sorting through differential affinities for substrate–chaperone complexes [42]. As in the flagellar T3SS, the crucial role of the cytoplasmic IM ring could be to act as a scaffold in the formation of the putative NF-T3SS C-ring. Indeed, pull-down assays have indicated an interaction between *S. flexneri* SctD-N and the C-ring component SctQ [39,41], whilst deletion of either *S. typhimurium* SctD-N or SctQ results in the formation of basal bodies lacking the needle appendage [31,41], indicating that these proteins are acting at a similar point in T3SS assembly. The structural flexibility of the adjoining cytoplasmic SctD-N ring [22^••^] and observed dynamic nature of the flagellar C-ring [43] could explain why the NF-T3SS C-ring has thus far remained elusive in EM reconstructions.

SctQ, the essential component of this sorting platform, shows sequence homology to the two flagellar C-ring components: FliM, which is of an equivalent size, and FliN, which encompasses the C-terminal third of the protein sequence. Recently, it has been discovered that in the pathogenic NF-T3SS background an internal initiation site within the gene enables this class of proteins to be produced as two alternative translational forms [44^•^,45^•^], a full length and a shorter C-terminal variant. Although a chaperone role for the shorter variant has been proposed [45^•^], these proteins could also be directly equivalent to FliM and FliN respectively as integral structural components of the C-ring, suggesting that the molecular arrangement of the putative NF-C-ring could be more similar to the flagellar C-ring than previously anticipated. Indeed, structures of the C-terminal portion of NF-T3SS SctQ [44^•^,46] show an intertwined dimeric assembly highly similar to that already observed for FliN [47], where each protomer is made of 5 β-strands, with the first and the second strand undergoing domain swapping within the dimer. This homodimer binds to monomeric full-length SctQ to form a 1:2 complex that could be acting as the building block of the putative NF-C-ring, in contrast to the proposed 1:4 stoichiometry of the flagellar FliM-FliN building block [44^•^]. Furthermore, in the current model of the flagellar T3SS, the C-ring is connected to the basal body by FliG [3] and thus it is tempting to speculate that the proposed interaction between the SctD-N ring and C-ring is indirect. Given the requirement for the SctK family in C-ring localization [48], and its ability to interact with SctQ, assignment of a FliG-like role to these homologues could be hypothesized [49].

Finally, the combination of crystal structures with electron cryotomography (ECT) has significantly advanced our understanding of the architecture of the T3SS EA and ATPase complex [23^••^]. The EA is made up of 5 integral IM proteins, with two of them (SctV and SctU) having globular C-terminal domains (SctV-C and SctU-C) protruding into the EA cytosol. It has been proposed to act as a secretion gate where substrates are gathered and uncoupled from their cognate chaperone to be routed out to the secretion path. Structurally the cytoplasmic domain of SctU is well characterized across species [50] and it is proposed to be mainly involved in export substrate selection in conjunction with SctP/FliK, a supposed molecular ruler for which structural information is only just becoming available [51]. Insights into the rest of the EA have advanced in the last few years. The structure of SctV-C is highly conserved in flagellar and non-flagellar homologues [52–55] and recently the crystal structure of the *S. flexneri* SctV-C demonstrated a homo-nonameric ring assembly [23^••^]. Crucially, not only did ring assembly mutations disrupt secretion, but variants containing mutations on the inner surface of the ring considerably affected protein export, suggesting that the route through the SctV ring is the path toward secretion. Fitting this structure into a torus of density in ECT maps of flagellar motors [56^••^] has begun the process of defining the geometry of the export machinery, placing the SctV-C ring midway between the IM and the ATPase complex (Figure 3). Interestingly, the presence of the SctV-C ring is independent of the ATPase and vice-versa. The ATPase complex is made of three soluble components, SctN, SctO and SctL, and structures of SctN/FliI [24,57] and SctO/FliJ [26^••^,58] reveal homology with the α/β and γ subunits of the F_0_F_1_-ATP synthase, respectively. Although previously characterized as a general chaperone, it has now been shown that SctO/FliJ has a coiled-coil structure that inserts into the central cavity of the ATPase hexamer, in a manner directly analogous to the ATP synthase, and that it is capable of rotation within a chimeric V_1_V_0_ A_3_B_3_J assembly [25]. In addition, the observed correlation between FliH length and ATPase/C-ring distance in flagellar motors [23^••^] combined with fluorescence data suggests that the ATPase complex is held underneath the SctV-C ring by SctL/FliH, which at its opposite end interacts with the hydrophobic patch at the dyad axis of the FliN dimer of the putative C-ring [59]. However, despite the recent progress in decoding the biophysical properties of its major components, the molecular architecture of the T3SS EA and C-ring clearly require further structural characterization in order to address mechanistic issues, such as the role of the protonmotive force [60], and localization of the crucial, low copy number components.

In summary, cumulative work over the last few years has highlighted the architectural similarities between the flagellar-T3SS and NF-T3SS. Intriguingly, especially in light of the ATPase complex structures, a recent study has suggested a proton-motive force dependent rotation of the NF-T3SS needle filament, thereby drawing further parallels to the flagellum [61^••^] and the latest work proposing a general mechanism for flagellar export [62^••^] may therefore also provide insight into the NF-T3SS. Future research concerning the molecular mechanisms of assembly and secretion by these nanomachines will undoubtedly provide further insight into the extent of the diversification of T3SSs.

## References and recommended reading

Papers of particular interest, published within the period of review, have been highlighted as:

• of special interest
•• of outstanding interest

## Figures and Tables

**Figure 1:**
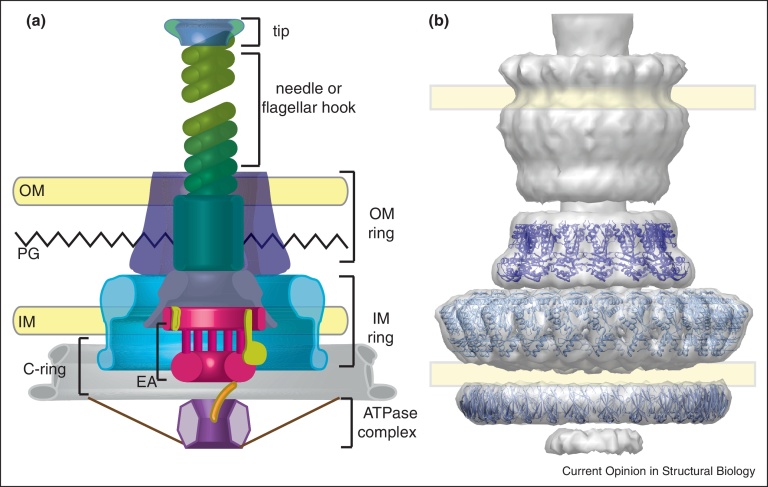
General architecture of the T3SS. **(a)** The cartoon schematizes the major subassemblies of the T3SS, showing their relative localization with respect to the outer membrane (OM), the peptidoglycan layer (PG) and the inner membrane (IM). **(b)** Surface view of the T3SS (C3) from *Salmonella typhimurium* (EMDB-1875; [22^••^]) with fitted atomic models [31] of SctC (PDB-3j1v), SctD-N (PDB-3j1w), SctD-C (PDB-3j1x).

**Figure 2:**
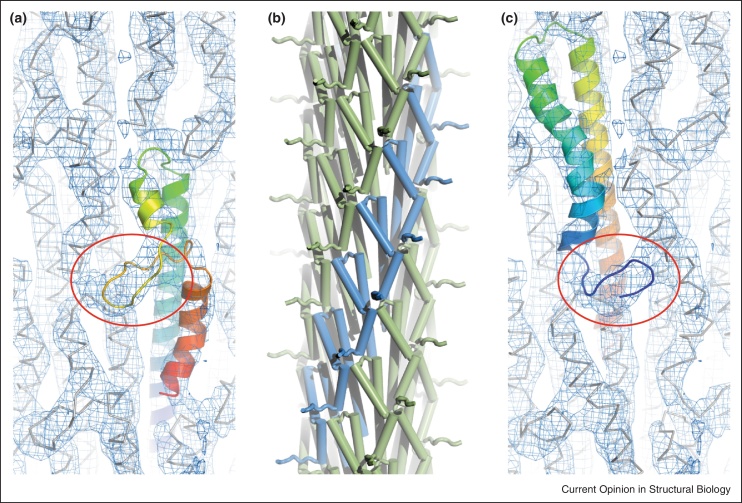
Alternate models for the helical needle assembly. **(a)** The high resolution EM map (EMDB-5352) and C-terminal out subunit fitting for the *Shigella flexneri* needle (PDB-3j0r; [15^•^]). **(b)** A cartoon representation of the *Salmonella enterica* needle with N-terminal out subunit built using Rosetta by Loquet *et al*. (PDB-2lpz; [16^••^]). **(c)** Trivial remodeling of the flexible N-terminus of the Loquet *et al*. [16^••^] subunit position demonstrates a good fit of this independently derived needle model to the Fuji *et al*. EM map. The circled density in (a) and (c) highlights the alternate interpretations of this region by either an unwound piece of the C-terminal helix [15^•^] or the flexible N-terminus (this work).

**Figure 3:**
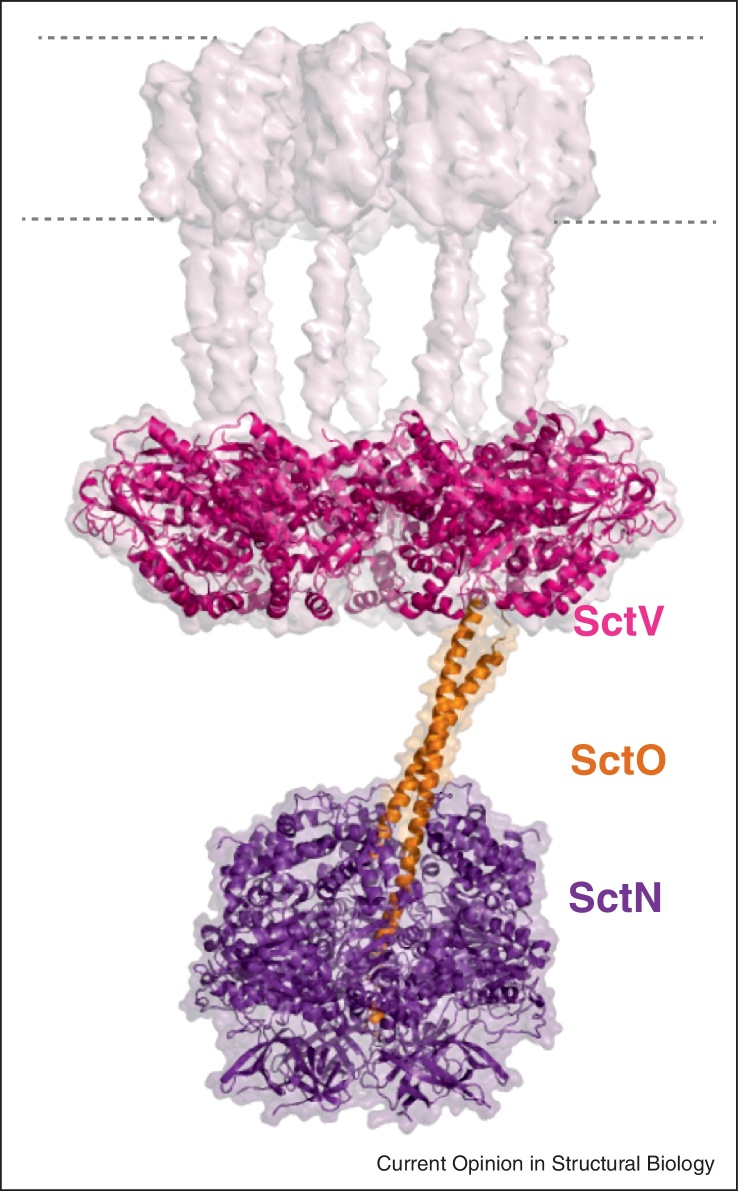
Geometry of the export machinery. The export machinery as built by Abrusci *et al*. [23^••^]. The SctV nonameric cage is represented as surface in light pink with fitted atomic model of the *Shigella flexneri* SctV-C (PDB-4a5p), in magenta. ATPase and its stalk are modeled using the atomic model form the *Escherichia coli* SctN (PDB-2obm) and the flagellar homologue of SctO form *Salmonella typhimurium* (PDB-3ajw) and colored in purple and orange, respectively.

**Table 1:** Summary of names of homologous proteins in different type three systems including the unified, Sct naming system

Functional name	Sct name	*Yersinia*	*Shigella*	*Salmonella* SPI-1	Flagellar homologue
Needle filament protein	SctF	YscF	MxiH	PrgI	–
Inner rod protein	SctI	YscI	MxiI	PrgJ	–
OM secretin ring	SctC	YscC	MxiD	InvG	–
IM outer ring	SctD	YscD	MxiG	PrgH	–
IM inner ring	SctJ	YscJ	MxiJ	PrgK	FliF
Minor export apparatus protein	SctR	YscR	Spa24	SpaP	FliP
Minor export apparatus protein	SctS	YscS	Spa9	SpaQ	FliQ
Minor export apparatus protein	SctT	YscT	Spa29	SpaR	FliR
Export apparatus switch protein	SctU	YscU	Spa40	SpaS	FlhB
Major export apparatus protein	SctV	YscV	MxiA	InvA	FlhA
Accessory cytosolic protein	SctK	YscK	MxiK	OrgA	FliG (?)
C-ring protein	SctQ	YscQ	Spa33	SpaO	FliM + FliN
Stator (ATPase regulator)	SctL	YscL	MxiN	OrgB	FliH
ATPase	SctN	YscN	Spa47	InvC	FliI
Stalk	SctO	YscO	Spa13	InvI	FliJ
Needle length regulator	SctP	YscP	Spa32	InvJ	FliK
